# Robotic Archwire Bending in Orthodontics: A Review of the Literature

**DOI:** 10.7759/cureus.56611

**Published:** 2024-03-20

**Authors:** Babitha Merin George, Siddarth Arya, Shwetha G S, Keerthana Bharadwaj, Vaishnavi N.S

**Affiliations:** 1 Orthodontics and Dentofacial Orthopaedics, RajaRajeswari Dental College & Hospital, Bengaluru, IND

**Keywords:** orthodontic treatment, bending apparatus, dental malocclusion, orthodontic archwires, robotics

## Abstract

Malocclusion is a widespread oral health issue that adversely affects individuals’ health and well-being. Currently, fixed orthodontics is considered the most efficient treatment for correcting malocclusion, with archwire bending playing a key role in orthodontic treatment. Traditionally, orthodontists manually performed archwire bending using various handheld pliers and other mechanical tools, requiring a significant amount of time, precision, and specialized training yet being unable to guarantee appliance accuracy. The process of shaping orthodontic wire is challenging due to its high stiffness and superelasticity, resulting in a time-consuming, laborious process that is prone to human errors. With advancements in orthodontics, traditional methods have taken a backseat, making way for innovative technologies that provide more accurate and personalized treatment options. The continuous efforts to enhance treatment efficiency, accuracy, efficacy, and patient experience have led to the integration of robotics into various orthodontic procedures. The use of robotics in archwire bending represents a breakthrough in orthodontics, offering unparalleled precision, consistency, and efficiency. This technology reduces treatment time and patient discomfort, overcoming the limitations of manual bending and enhancing orthodontic treatment overall. Hence, the present study aims to review the literature on robotic archwire bending in orthodontics, including their drawbacks and their impact on orthodontic treatment.

## Introduction and background

Malocclusion, a common type of oral disease resulting from genetic and environmental factors, is characterized by improper tooth alignment and irregular dental arches [1]. This condition affects mastication, pronunciation, appearance, and maxillofacial growth and can lead to oral disorders such as dental caries and periodontitis. The conventional orthodontic method is widely regarded as the most efficient technique for correcting malocclusion. In this treatment, the force produced by the distortion of the archwire is restored in order to straighten the misaligned teeth, thus making the archwire bending method a pivotal aspect of orthodontic treatment [2].

The traditional archwire bending technique relies heavily on the skill of the dentist, and the quality of the outcome is therefore dependent on their expertise. Additionally, varying cases require archwires with different parameters, necessitating multiple bends to achieve the optimal therapeutic result. Consequently, the archwire is prone to fatigue degradation and exhibits low bending efficiency [3]. Robotic technology can be used to bend orthodontic archwires, which effectively overcomes these shortcomings [4].

The Robot Institute of America defined a robot as “a reprogrammable multifunctional manipulator designed to move materials, parts, tools, or specialized devices through various programmed motions for the performance of a variety of tasks” [5]. Robots remain utilized in a wide range of tasks, including minimally invasive medical procedures, precise surgical operations, enhanced visual capabilities for specialists, and reduced recovery times for patients. Additionally, dentistry has seen significant advancements from traditional methods to modern technology, expanding the scope of dental treatment and systems [6]. This includes the ability to create full or partial dentures, place dental implants, and manipulate orthodontic archwire. The collaboration between technicians and specialists is integrated into the product framework [4]. Presently, the SureSmile system is regarded as the most cutting-edge archwire bending robot globally, with multifunctional capabilities such as mouth scanning and archwire bending [7]. Smith et al. [8] conducted an assessment of the experimental outcomes of the archwire for tooth tilt correction using this system. Zhang et al. [2] compared the pros and cons using finite element analysis and the mathematical method to describe the classical arch curve. The bending algorithm can offer an efficient control strategy for robotic orthodontic archwire bending when combined with the appropriate three-dimensional (3D) model [9].

In the current landscape of orthodontics, digital transformation has revolutionized the field, enabling the complete digitalization of data and 3D simulation for accurate diagnosis of patient-specific issues. Within the realm of robotics, this encompasses a wide array of applications, including the use of robots for precise X-ray imaging and positioning [10], tongue robots [11], mandibular [12] and condylar movement simulation robots [13,14], automated 3D cephalometric annotation [15], mandibular advancement appliances in obstructive sleep apnea patients [16,17], and bionic robots for stimulating the stomatognathic system [18,19].

The development of 3D imaging and assembly techniques facilitated the customization of orthodontic devices, thereby improving the efficacy of treatment. Technological advancements have resulted in the creation of two patient-specific products that utilize computer technology to generate a personalized treatment plan and fabricate a custom appliance, as exemplified by the Insignia system. Hence, the present study aims to review the literature on robotic archwire bending in orthodontics, including its drawbacks and their impact on orthodontic treatment.

## Review

The reviews for this article were sourced from PubMed, Google Scholar, the IEEE International Conference on Robotics and Automation, orthodontic journals, and professional associations.

Bending art system (BAS)

The initial computer-aided design and computer-aided manufacturing system specifically designed to produce custom orthodontic archwires was introduced in 1984 by Professor Helge Fischer-Brandies and his colleague in collaboration with an engineering company. The first prototype, known as BAS, was capable of producing orthodontic wires for both lingual and labial applications [20]. The system comprises three key components: a stereoscopic internal camera, a wire bending unit, and a computer program [21]. In 1998, the BAS software was enhanced with a force module to address concerns raised by Professor Helge Fischer-Brandies regarding the accurate estimation of forces acting on individual teeth [22].

BAS utilizes a series of custom-made archwires that are bent to the precise specifications required to move the teeth into their proper position. The BAS approach is designed to provide greater control and predictability in tooth movement, resulting in more efficient and effective orthodontic treatment. The process begins with a thorough examination and assessment of the patient’s teeth and jaw structure. Using advanced imaging technology, such as cone beam computed tomography (CBCT) and 3D scans, the orthodontist can create a detailed treatment plan tailored to the specific needs of the individual. This includes determining the exact positioning of the teeth and the degree of malalignment that needs to be corrected. Once the treatment plan is established, custom archwires are fabricated to match the unique specifications of the patient’s teeth. These archwires are then carefully bent to reflect the planned movement of the teeth.

The precise bending of the archwires is a key aspect of the BAS technique, as it allows for targeted and controlled forces to be applied to each tooth, guiding them into their desired positions. The custom archwires are periodically replaced throughout the treatment process to accommodate the changing alignment of the teeth. This ensures that the forces being applied to the teeth are continually optimized for maximum efficiency and efficacy. By utilizing custom-made archwires and advanced bending techniques, the BAS approach aims to minimize the duration of treatment and reduce the need for additional interventions.

**Figure 1:**
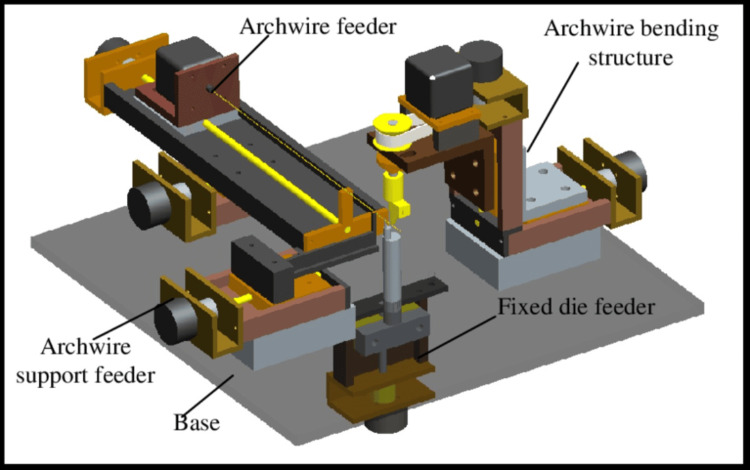
BAS BAS, Bending Art System Permission has been obtained from the original publisher for the re-publication of this figure [23].

One of the key advantages of the BAS technique is its ability to provide greater customization and precision in tooth movement. The use of custom archwires allows for more efficient force delivery, which can contribute to a more comfortable and less invasive orthodontic experience for the patient. Overall, the BAS represents a significant advancement in orthodontic treatment, offering a more personalized and targeted approach to tooth movement. By leveraging advanced technology and customized treatment planning, orthodontists can achieve more predictable and efficient results, ultimately leading to improved patient satisfaction and oral health outcomes (Figure 1).

LAMDA robotic wire bending system

**Figure 2:**
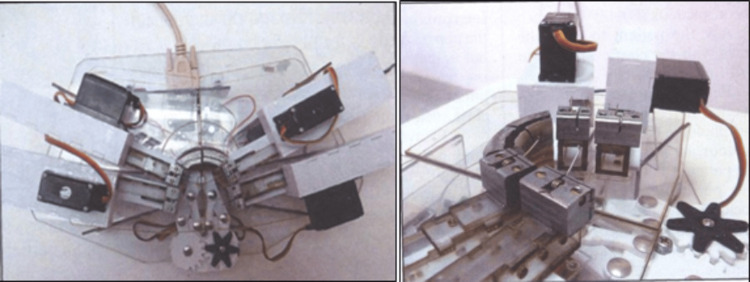
LAMBDA system Permission has been obtained from the original publisher for the re-publication of this figure [25].

The LAMDA system is a technology developed for the rapid and precise bending of archwires, utilizing a robotic apparatus capable of bending the archwire in two planes [24]. Introduced by Alfred Gilbert in 2011, the LAMDA program streamlines the manufacturing and bending process of archwires, focusing on the first dimensional order. The Hiro Bonding System, introduced by Hiro in 2008, handles the other two dimensions. By implementing an in-house wire bending robot, the need for external laboratories, associated costs, and waiting time for shipment of wires are eliminated, whether before or after bracket bonding (Figure 2).

Occlusal photographs are taken at each appointment to facilitate the use of LAMDA in determining the design and size of the next archwire. The four-engine robot (LAMBDA 1) phased out when the straight arch approach gained popularity in lingual orthodontics, as the arch no longer required correction between the canine and premolar and between the first and second molars.

The Lamdabot 2, equipped with 12 motors, is able to bend from one first molar to the first molar on the opposite side. This robot facilitates the manipulation of a straight archwire or a preformed wire, enabling the necessary bends to obtain the segmented orthodontic archwire. Like LAMBDA, this robot allows for the customization of orthodontic archwires.

Motoman UP6

The Motoman UP6 robot is utilized in orthodontic archwire bending and is equipped with a computer and an archwire-twisting device [26]. Due to the intricate shape of the archwire, flexibility is required at the robot’s end during the bending process. Moreover, the Motoman UP6 robot, which offers six degrees of freedom, meets the flexibility requirements necessary for the archwire bending task.

Cartesian type archwire bending robot

**Figure 3:**
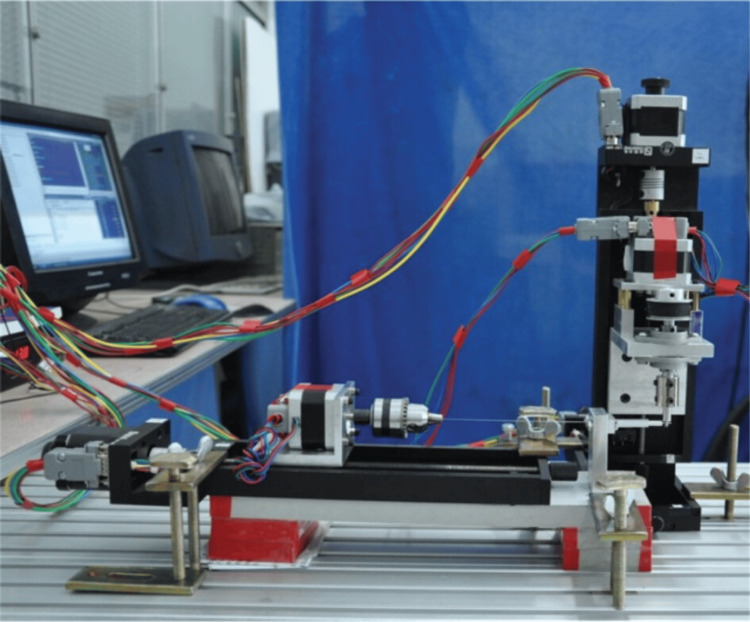
Cartesian type archwire bending robot Permission has been obtained from the original publisher for the re-publication of this figure [4].

The archwire bending system involves a Cartesian type robot comprising various components. These include the bending die, base, feed mechanism, archwire bending system, and the wire’s turning, feeding, and support structure (Figure 3) [27,28]. The bending process is examined, and the archwire’s structure is planned with the aid of associated software. Experimental studies are ongoing with the Cartesian setup, and the resulting data is being evaluated for comparison.

SureSmile system

In the realm of orthodontics, conventional braces typically rely on two-dimensional X-rays and plaster molds for treatment planning. On the other hand, the innovative SureSmile technique takes advantage of a cutting-edge 3D computer monitoring system, which enables the capture of exceedingly precise images of your teeth. This heightened level of precision empowers the orthodontist to devise an optimal series of movements for the teeth [28].

The SureSmile treatment commences with a meticulous scanning procedure, wherein a 3D computer model of the teeth is generated. This is accomplished through the use of a light scanner and CBCT. For subsequent processing, 3D models are then transmitted to the computer. The SureSmile archwire bending robot is the name of the twisting device. The twisting tool has a robot that is fixed to a base. A structure on the first grasping apparatus holds the archwire. It can either be fixed to a movable arm or fixed with respect to the base. The dentist inputs the position and necessary tension for the brackets and wires into the computer, which is then transmitted online to the SureSmile office [29].

Two robotic pliers then grasp an orthodontic wire and manipulate it by bending and twisting it to reposition the teeth as required [29]. Following this, the data is transmitted to a computer, where minor adjustments can be made. The dentist inputs data regarding the positioning and tension requirements for the brackets and wires into the system, which is then sent to the SureSmile head office. Subsequently, the robots come into play; two mechanical grasping pliers hold the archwire and mold it into the specified shape [28].

**Figure 4:**
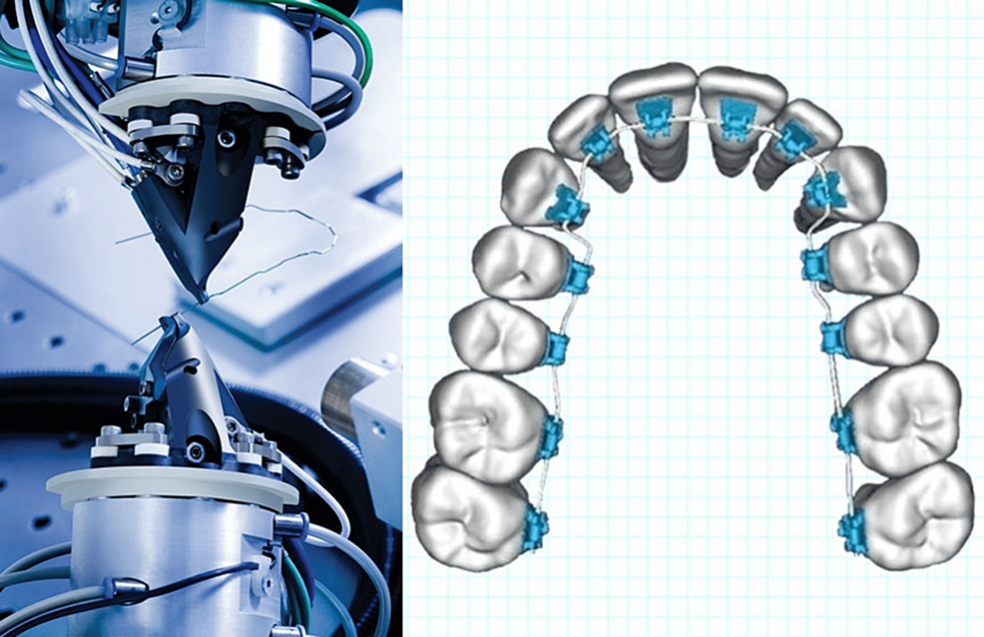
SureSmile system Permission has been obtained from the original publisher for the re-publication of this figure [31].

The second holding device is positioned at the far end of a movable six-axis robotic arm. The arm has a proximal portion that is fixed to the base and a distal end capable of relative motion around three rotational axes and three translational axes in relation to the stationary grasping apparatus. The optimal holding devices are equipped with constraint sensors to ascertain the required overbends for achieving the anticipated final shape of the archwire. In order to heat the wire and ensure the retention of its bent shape, they may integrate a resistive heating system, which involves applying current to the wire while it is held in a bent state (Figure 4) [30].

Insignia

**Figure 5:**
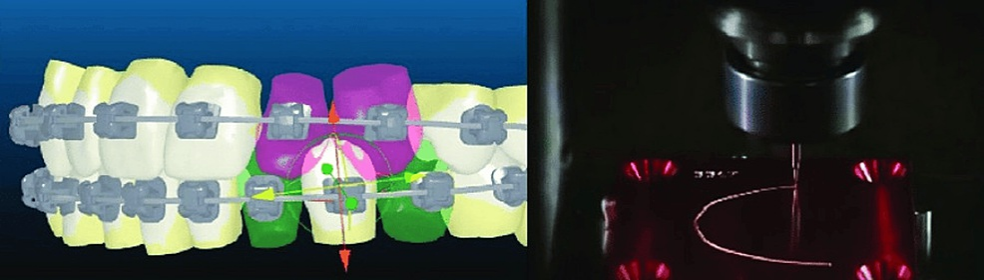
Insignia system Permission has been obtained from the original publisher for the re-publication of this figure [34].

Insignia, a remarkable bracket system, is the pinnacle of digital orthodontics, boasting an array of cutting-edge technologies. This system, unparalleled in its innovation, equips orthodontists to meticulously design and efficiently deliver impeccable results. The software enables them to plan and design the final occlusion, following which customized brackets and archwires are fabricated to reposition teeth according to the desired alignment. Utilizing a printing robot, the custom archwires are produced with an incredibly high level of precision and an exceedingly small margin of error, as provided by the system framework [32]. One of the advantages of Insignia is its ability to significantly reduce treatment time. Weber et al. have shown that patients treated with Insignia experienced a staggering 37% less treatment time compared to conventional methods. Moreover, Insignia offers the added advantage of reducing the number of office visits required on average (Figure 5) [33].

Others robotic archwire

The integration of the Robotics Operating System provides a robust framework for the control and coordination of the robot’s operations, allowing for seamless interaction between hardware and software components. The modular design of the hardware and software systems offers high dexterity and expandability, providing a versatile platform for automatic archwire bending [35].

An adaptive sampling-based planner is a key feature of the control system, responsible for generating bending plans for the robotic arm. This planner utilizes advanced algorithms to analyze the anticipated shape of the archwire and generate a sequence of bending actions that will achieve the specified configuration. The successful validation of the robot through simulation and physical experiments demonstrates its potential to revolutionize orthodontic practices, ultimately leading to improved treatment outcomes and patient experiences.

Discussion

In the field of orthodontics, there has been a continual effort to enhance the efficiency and effectiveness of appliances. Throughout its history, various modalities have been refined and advanced to maximize the quality of treatment. Recent studies evaluating the utilization of robots or automated machines to bend orthodontic archwires into the desired shapes have concluded that employing robots in contrast to conventional archwire manufacturing can enhance the reproducibility, efficiency, and overall quality of orthodontic treatment [26].

Moreover, robotic archwire bending has gained traction in various orthodontic procedures, with techniques such as Insignia and SureSmile emerging as widely recognized methods that harness the capabilities of robots in archwire manipulation.

The adoption of customized brackets manufactured by robots has shown promise for enhancing the effectiveness and efficiency of orthodontic treatment. By addressing individual tooth morphology variations and enabling precise virtual planning of individual tooth movements, this approach has demonstrated the potential to improve treatment outcomes [32,33,35-39]. The highest level of technological readiness has been reflected in the successful implementation of these systems in end-use operations, highlighting the feasibility and practicality of incorporating robotic manufacturing techniques in orthodontic treatment protocols [40-42].

According to Gilbert et al., the archwire bending robot demonstrated markedly higher average accuracy scores in comparison to proficient orthodontic practitioners who were tasked with the same assignments as the robot [30]. This suggests that robotic archwire bending offers a quick and precise approach that has the potential to shorten treatment time and enhance patient comfort, ultimately leading to improved treatment outcomes. These results position the use of robots for archwire bending as not only a viable but also a potentially more effective alternative to conventional methods.

Studies have also explored the impact of utilizing specialized robots to bend the archwire on treatment duration. While some findings have indicated a significant reduction in treatment time through the use of robotic archwire bending, it is noteworthy that this outcome was not universally supported. This is because studies examining the effect of robotic archwire bending on treatment duration were primarily conducted on mild cases and did not encompass severe malocclusions.

Drawbacks

The unique characteristics of the archwire and the intricate nature of the oral environment pose challenges for robots aiming to meet the requirements for orthodontic treatment success. While robots are designed to be flexible, reliable, and precise, efforts must focus on reducing the degree of freedom of robots. Human and computer collaboration is pivotal for advancements, and future research should emphasize the 3D virtual depiction of orthodontic devices and virtual treatment prediction. Research endeavors should prioritize the development of spring-back and bending algorithms for archwire bending robots. Additional research is necessary to investigate the physical properties of orthodontic archwires in order to improve the precision of archwire bending robots. This research should focus on reducing the degree of freedom of the robots [26].

## Conclusions

Utilization of robotics or machines for twisting the archwire in fixed orthodontic appliances can lead to substantially improved treatment outcomes and a strikingly shortened treatment time compared to conventional techniques reliant on human expertise. The precision and accuracy offered by robotic manipulation, coupled with decreased patient discomfort, demonstrate the potential for transformative enhancements in orthodontic care. The widespread recognition of procedures such as Insignia and SureSmile, which leverage robotic technologies in archwire twisting, underscores the growing prominence and acceptance of robotic-assisted orthodontic methods.
